# Specific topics, specific symptoms: linking the content of recurrent involuntary memories to mental health using computational text analysis

**DOI:** 10.1038/s44184-023-00042-x

**Published:** 2023-12-18

**Authors:** Ryan C. Yeung, Myra A. Fernandes

**Affiliations:** 1 Department of Psychology, University of Waterloo, Waterloo, ON Canada; 2 Rotman Research Institute, Baycrest Health Sciences, Toronto, ON Canada

**Keywords:** Human behaviour, Anxiety, Depression, Post-traumatic stress disorder

## Abstract

Researchers debate whether recurrent involuntary autobiographical memories (IAMs; memories of one’s personal past retrieved unintentionally and repetitively) are pathological or ordinary. While some argue that these memories contribute to clinical disorders, recurrent IAMs are also common in everyday life. Here, we examined how the content of recurrent IAMs might distinguish between those that are maladaptive (related to worse mental health) versus benign (unrelated to mental health). Over two years, 6187 undergraduates completed online surveys about recurrent IAMs; those who experienced recurrent IAMs within the past year were asked to describe their memories, resulting in 3624 text descriptions. Using a previously validated computational approach (structural topic modeling), we identified coherent topics (e.g., “Conversations”, “Experiences with family members”) in recurrent IAMs. Specific topics (e.g., “Negative past relationships”, “Abuse and trauma”) were uniquely related to symptoms of mental health disorders (e.g., depression, PTSD), above and beyond the self-reported valence of these memories. Importantly, we also found that content in recurrent IAMs was distinct across symptom types (e.g., “Communication and miscommunication” was related to social anxiety, but not symptoms of other disorders), suggesting that while negative recurrent IAMs are transdiagnostic, their content remains unique across different types of mental health concerns. Our work shows that topics in recurrent IAMs—and their links to mental health—are identifiable, distinguishable, and quantifiable.

## Introduction

Memories of the personal past that are retrieved unintentionally and effortlessly have been termed involuntary autobiographical memories (IAMs^1^). Recent evidence suggests that some IAMs are experienced *recurrently*—that is, episodes of the same event can be retrieved repetitively and involuntarily^2^. Past studies indicate that recurrent IAMs are commonly experienced in everyday life: large proportions (52–55%) of general populations (e.g., undergraduates, nationally representative samples, community-dwelling older adults) have endorsed experiencing at least one recurrent IAM within the past year^2–4^. However, recurrent IAMs have also been conceptualized as harmful or characteristic of psychiatric disorders^5,6^, despite their prevalence among the public. For instance, these memories have been described as a transdiagnostic component of many clinical disorders (e.g., depression, anxiety, PTSD), acting as a mechanism by which psychopathology emerges or is maintained^5,7^. As such, recurrent IAMs have been simultaneously characterized as maladaptive or clinically relevant on one hand^5^ and benign or pleasant on the other^2^.

This discrepancy has been acknowledged by some researchers, who have suggested that while many people experience recurrent IAMs, it may be the case that only some subset of IAMs are dysfunctional or related to poor mental health^8–11^. Indeed, our recent work has supported these hypotheses, finding that the maladaptive subset of recurrent IAMs could be those that are negative in valence: participants who experienced recurrent IAMs with self-reported negative valence had elevated symptoms of depression, PTSD, social anxiety, and general anxiety compared to those who experienced neutral, positive, or no recurrent IAMs^3,4^. However, solely relying on self-reported ratings of memories’ phenomenological properties (e.g., valence) is a major limitation of this field to date. For one, valence ratings are confounded with content (i.e., what people report remembering) since certain events are likely to be more negative or positive than others. Without examining the events that participants are remembering, we cannot conclude as to whether it is negative valence per se that characterizes maladaptive recurrent IAMs or that maladaptive recurrent IAMs tend to involve certain types of content. Prior attempts to characterize recurrent IAMs have been done without knowing what these memories are actually about, prompting us to ask if content analysis could help explain differences between maladaptive and benign recurrent memories.

Past content analyses of autobiographical memories have been fruitful at describing the events that participants remember, revealing common topics such as accidents, holidays, and interpersonal relationships^12–15^. Importantly, some suggest that content in recurrent IAMs might change as a function of mental health status, and could even provide insights into how recurrent IAMs might diverge across different disorders^5,6,16–18^. In other words, researchers have hypothesized that content in AMs (especially recurrent IAMs) differs between (1) those experiencing high versus low levels of psychopathology, as well as between (2) those with different mental health disorders (disorder-specific content). For example, early work has reported that recurrent IAMs about abuses or assaults were significantly related to greater depression severity, whereas recurrent IAMs about other topics (e.g., “illness or death”, “relationships/family”) were not significantly related to psychopathology^19^. More generally, some studies have found that AM content differed between those high versus low in symptoms, or with versus without diagnoses: patients with severe health anxiety have been more likely to report AMs about disease, illness, or death compared to participants without diagnoses^20^, and participants with high social anxiety have been more likely to report social anxiety-related AMs than participants with low social anxiety^21^. Researchers have also observed linguistic differences in AMs produced by individuals with versus without psychiatric diagnoses. For instance, participants with social anxiety disorder have been found to use more self-referential, anxiety-related, and sensory language compared to nonpsychiatric controls^22^. Beyond distinguishing between high versus low levels of psychopathology, these content differences in AMs have also been theorized to be disorder-congruent, in that recurrent IAMs should differ across disorders in terms of their themes or topics^5^. Indeed, linguistic variables (e.g., sensory-somatic and self-referential language) have been shown to differentiate AMs produced by those with bipolar disorder, unipolar depression, or no diagnoses^23^.

Though these results have supported that there are associations between AM content and mental health, numerous studies have not reached such conclusions. Other researchers have observed no significant differences in IAM content as a function of psychopathology, including between dysphoric versus nondysphoric participants^24^ and between participants with high versus low social anxiety^25^. Previous work has also failed to find evidence of disorder-congruent content: while AM content from participants with anxiety disorders was significantly different from nonclinical controls, content did not significantly differ across various disorders (i.e., social anxiety disorder versus panic disorder^26^). Taken together, it remains inconclusive as to whether AM content varies across symptom severity or across disorders. It is possible that some of these mixed findings could have arisen due to limitations such as (1) relatively small sample sizes, (2) modeling content as pure categories (e.g., labeling each memory as containing a single topic), and (3) valence being entangled with content.

First, sample sizes have typically been relatively small (<100) due to the populations being studied (e.g., people with psychiatric diagnoses), which limits recruitment. Sample sizes also have an upper limit when conducting traditional, manual content analyses, since large volumes of text can quickly become unfeasible to code manually. While data from relatively small sample sizes have provided valuable foundations for this research area, it is yet to be seen whether past findings generalize to larger, nonclinical samples. Second, the prevailing method of content analysis—single, mutually exclusive content labels being assigned to each document (i.e., single-membership models)—is a rather coarse measure of content. Evidence suggests that mixed-membership models (i.e., assuming that each document is a *mixture* of topics) offer a more granular measure of content that can illustrate topic structures distinct from those produced by single-membership models^27,28^. Third, past studies have not typically disentangled content and valence. We believe examining both content and valence simultaneously is an important open research question because while some types of content likely involve congruent valence (e.g., deaths and negative valence), content and valence can also be relatively independent. A participant might recall a relatively unpleasant event yet ascribe neutral or positive valence to the memory (e.g., failing a test, which subsequently led to improvements in study habits); conversely, a memory involving a relatively mundane event can feel highly distressing (e.g., a family dinner, which evokes feelings of homesickness after having moved out). Some have suggested that content is relatively unimportant compared to feelings and thoughts evoked by one’s IAMs. For example, individuals’ negative appraisals of their IAMs have been found to be better predictors of depression symptoms than experimenter-rated severity of the recalled event^29^. Given this, we asked whether recalled details of the event still matter after accounting for the valence ascribed to the memory. While many previous studies have measured content and valence in AMs and incorporated these variables into their analyses (e.g., matching AMs for emotional intensity^22^), to the best of our knowledge, studies to date have not simultaneously examined the relationships of IAM content and valence with psychopathology.

Here, we tested the hypothesis that content in recurrent IAMs would be associated with symptoms of mental health disorders, unique from previously observed links between self-reported valence and psychopathology^3,4^. Further, we also asked whether any relationships between content and symptoms would be distinct across different disorders (e.g., disorder-specific content). To address these questions, we analyzed both content and self-reported valence in recurrent IAMs experienced by a large nonclinical sample.

## Methods

In a previous study, we conducted the first large-scale content analysis of recurrent IAMs using computational techniques (e.g., machine learning, natural language processing) and highlighted the validity of using semi-automated methods for content analysis^15^. Here, we used the same approach to assess how symptoms of mental health disorders (i.e., depression, posttraumatic stress, social anxiety, general anxiety) might uniquely predict the use of different content categories (i.e., topics) within recurrent IAMs. By using computational methods, the current study analyzed data from a much larger sample size (*N* = 6187) than previous work and allowed us to ask more nuanced questions about content in recurrent IAMs (e.g., modeling topics as continuous variables rather than categorizing memories as containing single topics).

### Participants

As part of a previous study^15^, a convenience sample of undergraduate students was recruited at the University of Waterloo, who participated in return for course credit. Data were collected in five waves between September 2018 and February 2020, with each wave occurring at the start of an academic term (i.e., Fall/September, Winter/January, Spring/May). In total, 6187 unique individuals participated, and they produced 3624 text responses (not all participants experience recurrent IAMs, so not all participants can produce text responses; see Recurrent Memory Scale under Materials). Of these participants, 71% were women, 28% were men, and 1% were nonbinary, genderqueer, or gender nonconforming. Participants were mostly White/Caucasian (39%), East Asian (23%), or South Asian (19%), and were primarily born in Canada (66%), China (9%), or India (5%). Mean age was 19.9 (*SD* = 3.3, range = 16–49).

### Materials

#### Recurrent Memory Scale

The Recurrent Memory Scale^3^ was used to assess participants’ recurrent IAMs. Participants indicated if they had experienced at least one recurrent IAM within the past year, not within the past year, or never^2^. If they had experienced at least one within the past year, they wrote a brief description of their one most frequently recurring IAM and rated it on ten 5-point Likert scales (e.g., frequency of recurring, valence^3^). For instance, valence of their most frequently recurring IAM was assessed using the item “Is the recollection emotionally very positive, positive, neutral, negative, or very negative?” (−2 = *very negative*, 0 = *neutral*, 2 = *very positive*). While participants were administered the full scale, here we focus on participants’ text descriptions of their recurrent IAMs (content) and self-reported valence ratings.

#### Depression Anxiety Stress Scales

The Depression Anxiety Stress Scales-21 (DASS-21^30^) consists of 21 items with three subscales: depression (DASS-D), anxiety (DASS-A), and stress (DASS-S). Internal consistency was high in the current sample for the full scale (*α* = .95) and the subscales for depression (*α* = .91), anxiety (*α* = .87), and stress (*α* = .89).

#### Posttraumatic Stress Disorder Checklist for DSM-5

The Posttraumatic Stress Disorder Checklist for DSM-5 (PCL-5^31^) consists of 20 items assessing symptoms of PTSD. Participants indicated the degree to which they experienced symptoms in the past month following any very stressful event of their choosing. Internal consistency was high in the current sample (*α* = .96).

#### Social Phobia Inventory

The Social Phobia Inventory (SPIN^32^) consists of 17 items assessing fear, anxiety, and physical discomfort experienced during social situations. Internal consistency was high in the current sample (*α* = .95).

#### State-Trait Inventory of Cognitive and Somatic Anxiety—Trait Version

The State-Trait Inventory of Cognitive and Somatic Anxiety—Trait Version (STICSA-T^33^) consists of 21 items assessing cognitive and somatic aspects of trait anxiety. Internal consistency was high in the current sample (*α* = .94).

### Procedure

Participants opted into completing this study, for which they received course credit towards undergraduate psychology courses. It consisted of a 60-minute online survey completed in a single session. This survey was used by the University of Waterloo’s Department of Psychology to characterize students volunteering to participate in psychology-related studies. After providing informed consent, participants voluntarily completed a battery of questionnaires in a randomized order, including the Recurrent Memory Scale and mental health indices (DASS-21, PCL-5, SPIN, and STICSA-T). All other measures in the online survey were unrelated to the current study (e.g., administered by other researchers at the University of Waterloo). All procedures were approved by the University of Waterloo’s Office of Research Ethics (Protocol #40049).

### Data preparation

Prior to analysis, we first used supervised machine learning (ML) to detect and remove invalid texts; these include “don’t know” responses, incomprehensible or nonsensical responses, or responses that are irrelevant to the question (e.g., describing dreams when the question asked about memories^34^). Removing invalid texts is a recommended step in text analysis^35,36^ because invalid texts are unrelated to the construct in question (here, recurrent IAMs), and excluding them reduces noise in the data. Previous work has confirmed that ML-based methods can be more effective at identifying invalid text responses compared to other existing approaches, such as response length or time^34^. Here, the ML-based approach identified 202 texts as invalid (71 human labeled, 131 model predicted^15^), all of which were excluded from further analyses.

Valid texts were then preprocessed following current recommendations^35–37^, including tokenization, cleaning, stop word removal^38^, vocabulary pruning^39^^,^ and lemmatization^40^. Texts were represented using a bag-of-words, unigram approach^41^, which decomposes texts into singular words without retaining information about word order.

### Topic modeling

We discovered topics in participants’ descriptions of their recurrent memories using structural topic modeling (STM^42,43^). STM is a method of unsupervised machine learning that estimates hidden topic structures that could have plausibly produced the observed set of documents (i.e., corpus). By using texts as the input, topic modeling can output topics, or groups of words that can be interpreted as themes in the input texts^42,44,45^. Given the success of past work highlighting STM as a valid method of semi-automated content analysis with AM texts (see^15^ for details about preprocessing, model selection, and validation with human judgment), we extended our prior approach to answer novel questions about recurrent IAMs and psychopathology.

In previous work, we constructed topic models based on this dataset using only one covariate: participants’ self-reported ratings of the memory’s valence^15^. Here, we analyzed this dataset in conjunction with mental health-related covariates: participants’ current symptoms of depression (DASS-D), PTSD (PCL-5), social anxiety (SPIN), and general anxiety (STICSA-T). Data and code supporting the findings of this study are openly available on the Open Science Framework (10.17605/OSF.IO/GUR5V).

## Results

### Recurrent IAM valence predicts symptoms of mental health disorders

**Fig. 1:**
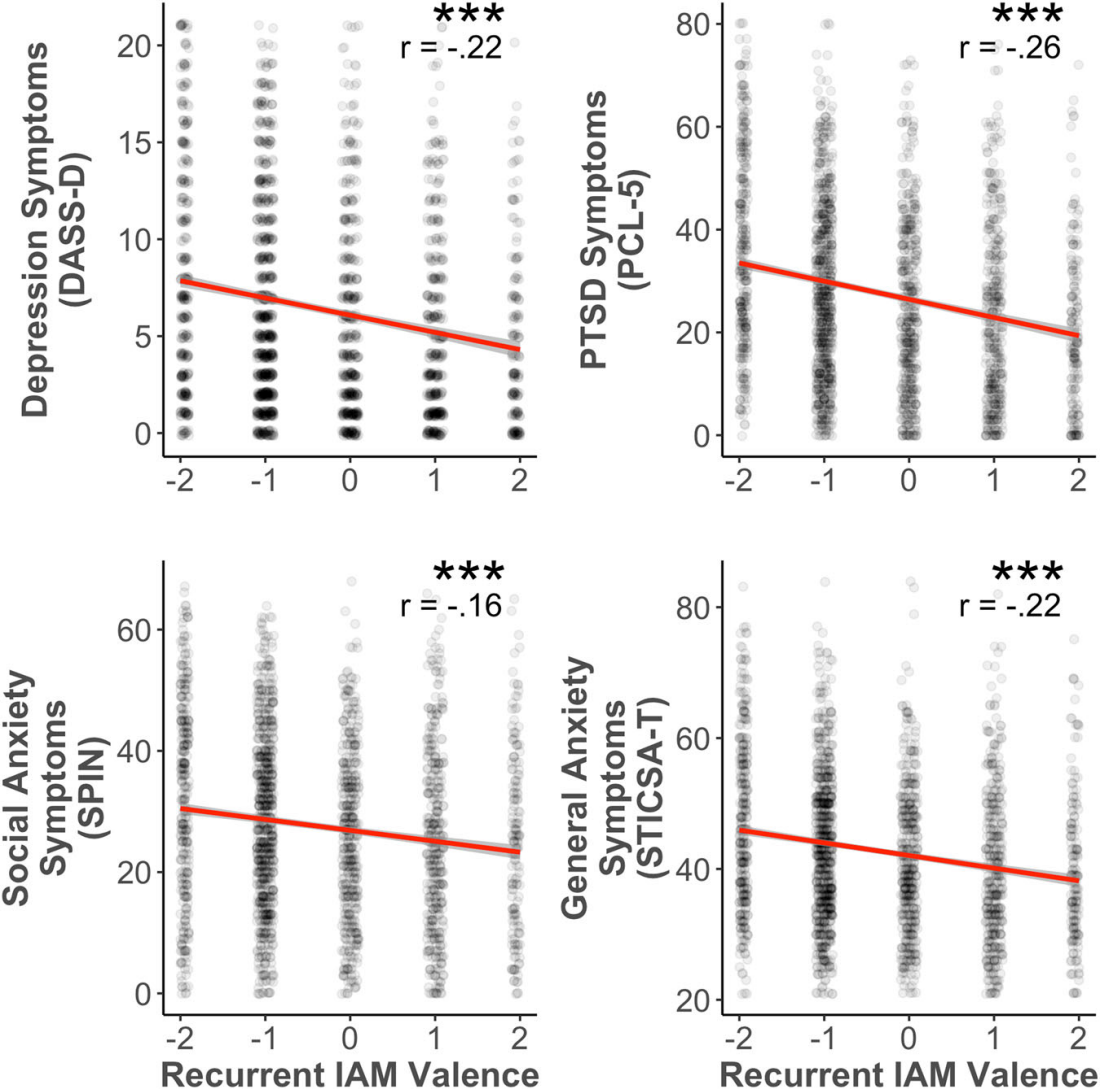
Self-reported valence ratings of recurrent IAMs and symptoms of mental health disorders. *Note*. DASS-D Depression, Anxiety, Stress Scales—Depression Subscale, PCL-5 PTSD Checklist for DSM-5, SPIN Social Phobia Inventory, STICSA-T State-Trait Inventory of Cognitive and Somatic Anxiety—Trait Version, IAM involuntary autobiographical memory. Shaded ribbons represent 95% confidence intervals. ***p < .001.

We replicated our previous findings in that negative valence in recurrent memories was significantly related to greater symptoms of depression, PTSD, social anxiety, and general anxiety^3,4^ (Fig. 1). Elevated symptoms were associated with more negative valence in recurrent IAMs. Correlations between recurrent IAM valence and symptoms of mental health disorders were all significant (*p*s < .001, *r*s < −.16).

As an exploratory analysis, we also examined whether these patterns held when excluding participants who scored above clinically relevant cutoffs on any of the mental health indices^31,46–48^ (Fig. S1 in Supplementary materials). Even following these exclusions (*n*_excluded_ = 1562; 63%), all patterns remained significant (*p*s < .03, *r*s < −.07).

### Topic structure and modeling

To examine content, we implemented structural topic modeling (STM) using the {stm} package in R^43^. Model selection and validation procedures are reported elsewhere in detail^15^. In brief, researchers must select an a priori number of topics to be identified when using STM^43^. To select an appropriate number of topics, we simulated and inspected models with the same parameters (e.g., covariates) across a varying number of topics (5–25 topics^36,49^). We then selected a final number of topics using a two-stage approach^41,50^. First, internal validation (based on computed metrics derived from the data) guided the initial selection of three candidate models out of the twenty simulated models. Second, external validation (based on human judgment and performance measures) guided the selection of the final model out of the three candidate models^15^. Previous work has indicated consistency between the current topics (discovered via semi-automated methods) and topics typically found in AMs (using manual methods^15^). The final topic structure obtained is shown in Table 1. Inclusion of the additional mental health-related covariates (participants’ scores on the DASS-D, PCL-5, STICSA-T, and SPIN) did not alter the topic structure obtained during the original study^15^, which only included valence as a covariate. Correlations between topics are also described in this previous study^15^.

**Table 1:** Topics in recurrent IAMs.

Topic Number	Researcher-Assigned Label	Top Ten Most Representative Words (Based on FREX Scores)	Topic Prevalence (%)
1	Stressful events	sad, forget, hospital, feel, without, guilt, anxious, relive, try, strong	5.6
2	Negative past relationships	relationship, negative, situation, involve, traumatic, previous, past, experience, emotion, similar	10.1
3	Physical activities and performance	game, play, enjoy, song, dance, listen, performance, music, soccer, around	4.2
4	Embarrassing events	fear, elementary, moment, set, embarrassing, work, peer, embarrass, along, able	5.1
5	Close relationships	spend, together, boyfriend, first, breakup, break, interaction, girlfriend, rude, cheat	6.4
6	Illnesses, injuries, and deaths	ago, accident, away, member, year, pass, last, family, drive, car	5.7
7	Confrontations, fights, and arguments	attack, fight, lose, argue, argument, end, parent, realize, unable, leave	6.3
8	Abuse and trauma	assault, abuse, date, ex, sexually, dream, significant, trauma, fail, seem	4.7
9	Conversations	someone, conversation, say, person, something, else, appear, fact, message, tell	6.3
10	Environments and locations	street, trip, walk, house, light, outside, hit, cat, travel, dad	5.8
11	Interactions with friends	friend, group, another, mine, talk, good, take, boy, highschool, chat	7.4
12	Communication and miscommunication	question, ask, teacher, class, guy, high, answer, send, interview, put	5.9
13	Subjective experiences of retrieval	frequent, childhood, mind, recollection, pop, recurrently, random, recent, sometimes, come	8.7
14	Subjective descriptions of detailed and/or time-specific recollections	specific, can, part, child, detail, stuff, nothing, remember, study, suddenly	5.3
15	Experiences with family members	watch, mom, sister, grandma, vacation, movie, eat, cousin, park, home	8.5
16	Reflections on decisions	get, like, just, every, back, etc, life, happy, thing, regret	3.9

*FREX* frequency-exclusivity (Bischof & Airoldi, 2012).

### Predicting topic prevalence using symptoms of mental health disorders

Symptoms of mental health disorders significantly accounted for unique variance in topic prevalence, even when controlling for valence ratings and symptoms of all other disorders (see 10.17605/OSF.IO/GUR5V for details). We found unique relationships between specific topics and specific symptoms of disorders, above and beyond how positive or negative a memory was rated (Table 2).

**Table 2:** Significant predictors of topic prevalence in recurrent IAMs

Topic #	Predictor	*B*	*SE* *B*	*p*
1	Valence	−0.015	0.0016	<.001
2	Valence	−0.019	0.0024	<.001
2	PCL-5	0.00069	0.00025	.006
2	SPIN	−0.00082	0.00026	.002
3	Valence	0.019	0.0017	<.001
4	PCL-5	−0.00044	0.00018	.02
5	Valence	0.012	0.0020	<.001
7	Valence	−0.022	0.0020	<.001
8	Valence	−0.010	0.0017	<.001
8	DASS-D	0.0016	0.00060	.009
8	SPIN	−0.00039	0.00017	.03
9	Valence	−0.014	0.0016	<.001
9	PCL-5	−0.00042	0.00017	.01
9	STICSA-T	0.00074	0.00030	.01
10	Valence	0.0086	0.0023	<.001
11	Valence	0.0061	0.0018	<.001
11	PCL-5	−0.00037	0.00019	.049
12	Valence	−0.011	0.0021	<.001
12	SPIN	0.00054	0.00026	.04
13	Valence	0.012	0.0020	<.001
15	Valence	0.031	0.0024	<.001
16	Valence	0.0052	0.0010	<.001
16	SPIN	0.00027	0.00011	0.02

Valence refers to participants’ self-reported ratings of their recurrent memories (−2 = *very negative*, 0 = *neutral*, 2 = *very positive*). Only significant predictors are presented here; if a predictor is absent for a topic, it was nonsignificant (see 10.17605/OSF.IO/GUR5V for all predictors and additional details).

*DASS-D* Depression, Anxiety, Stress Scales—Depression Subscale, *PCL-5* *PTSD* Checklist for DSM-5, *SPIN* Social Phobia Inventory, *STICSA-T* State-Trait Inventory of Cognitive and Somatic Anxiety—Trait Version.

#### Depression

**Fig. 2:**
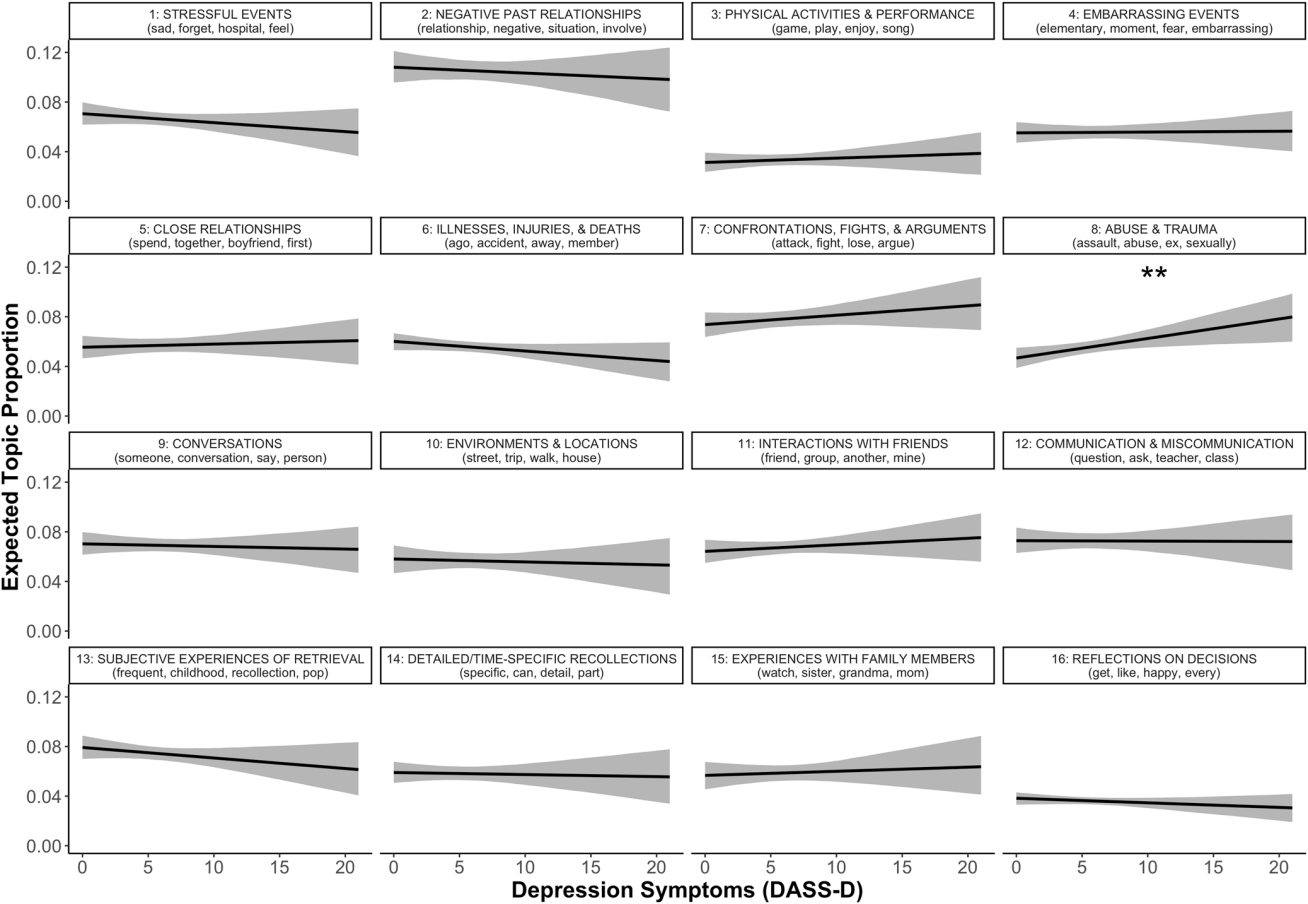
Predicted topic prevalence using depression symptoms. DASS-D Depression, Anxiety, Stress Scales—Depression Subscale. Different panels represent different topics, denoted by topic numbers and most representative words at the top of each panel. **p < .01. Shaded ribbons represent 95% confidence intervals.

Depression symptoms were significantly and uniquely predictive of greater use of topic 8 (“Abuse and trauma”; Fig. 2).

#### PTSD

**Fig. 3:**
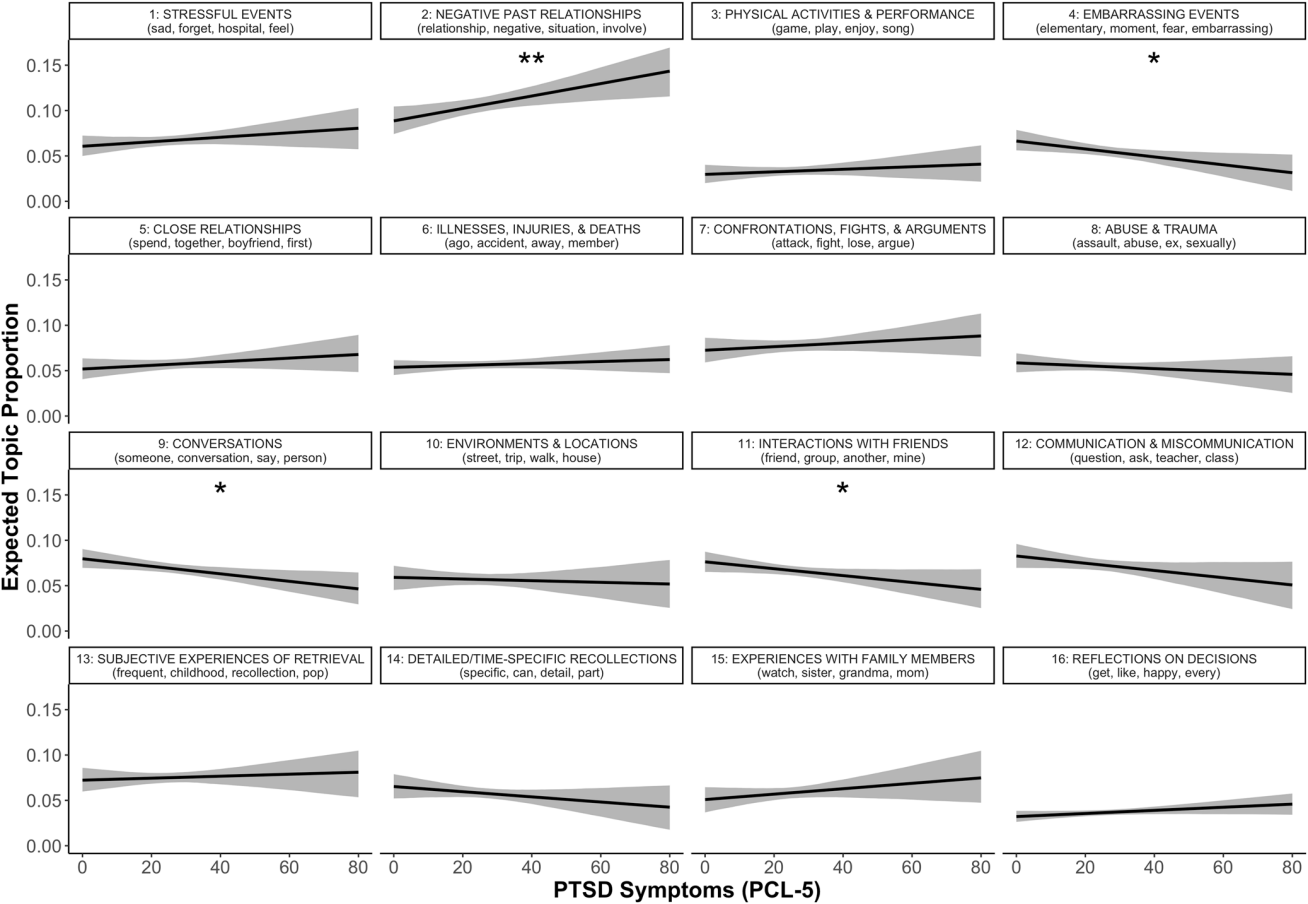
Predicted topic prevalence using PTSD symptoms. PCL-5 = PTSD Checklist for DSM-5. Different panels represent different topics, denoted by topic numbers and most representative words at the top of each panel. **p < .01, *p < .05. Shaded ribbons represent 95% confidence intervals.

PTSD symptoms were significantly and uniquely predictive of greater use of topic 2 (“Negative past relationships”). Further, PTSD symptoms were significantly and uniquely predictive of less use of topic 4 (“Embarrassing events”), topic 9 (“Conversations”), topic 11 (“Interactions with friends”; Fig. 3).

#### Social anxiety

**Fig. 4:**
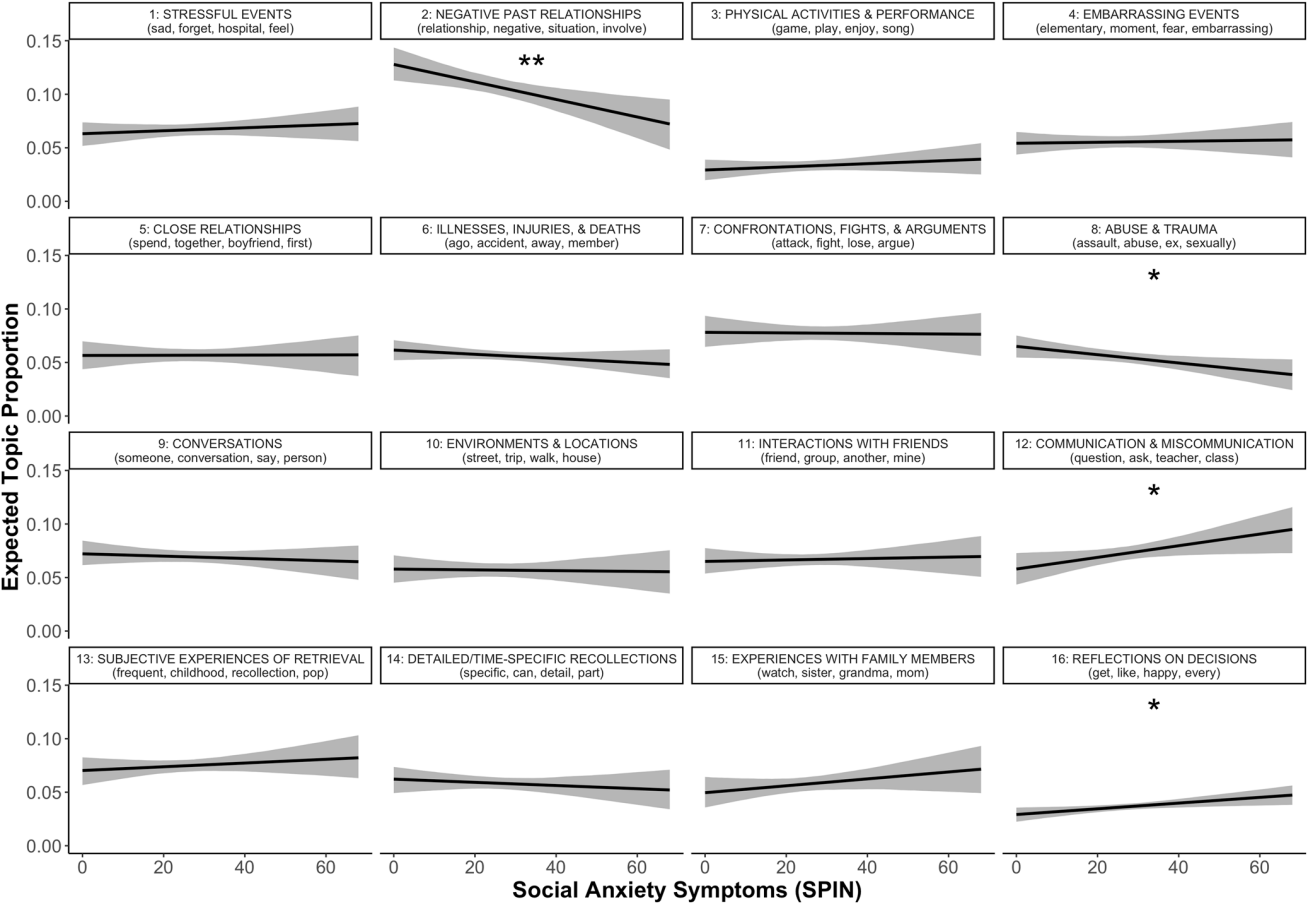
Predicted topic prevalence using social anxiety symptoms. SPIN Social Phobia Inventory. Different panels represent different topics, denoted by topic numbers and most representative words at the top of each panel. **p < .01, *p < .05. Shaded ribbons represent 95% confidence intervals.

Social anxiety symptoms were significantly and uniquely predictive of greater use of topic 12 (“Communication and miscommunication”) and topic 16 (“Reflections on decisions”). Further, social anxiety symptoms were significantly and uniquely predictive of less use of topic 2 (“Negative past relationships”), topic 8 (“Abuse and trauma”; Fig. 4).

#### General anxiety

**Fig. 5:**
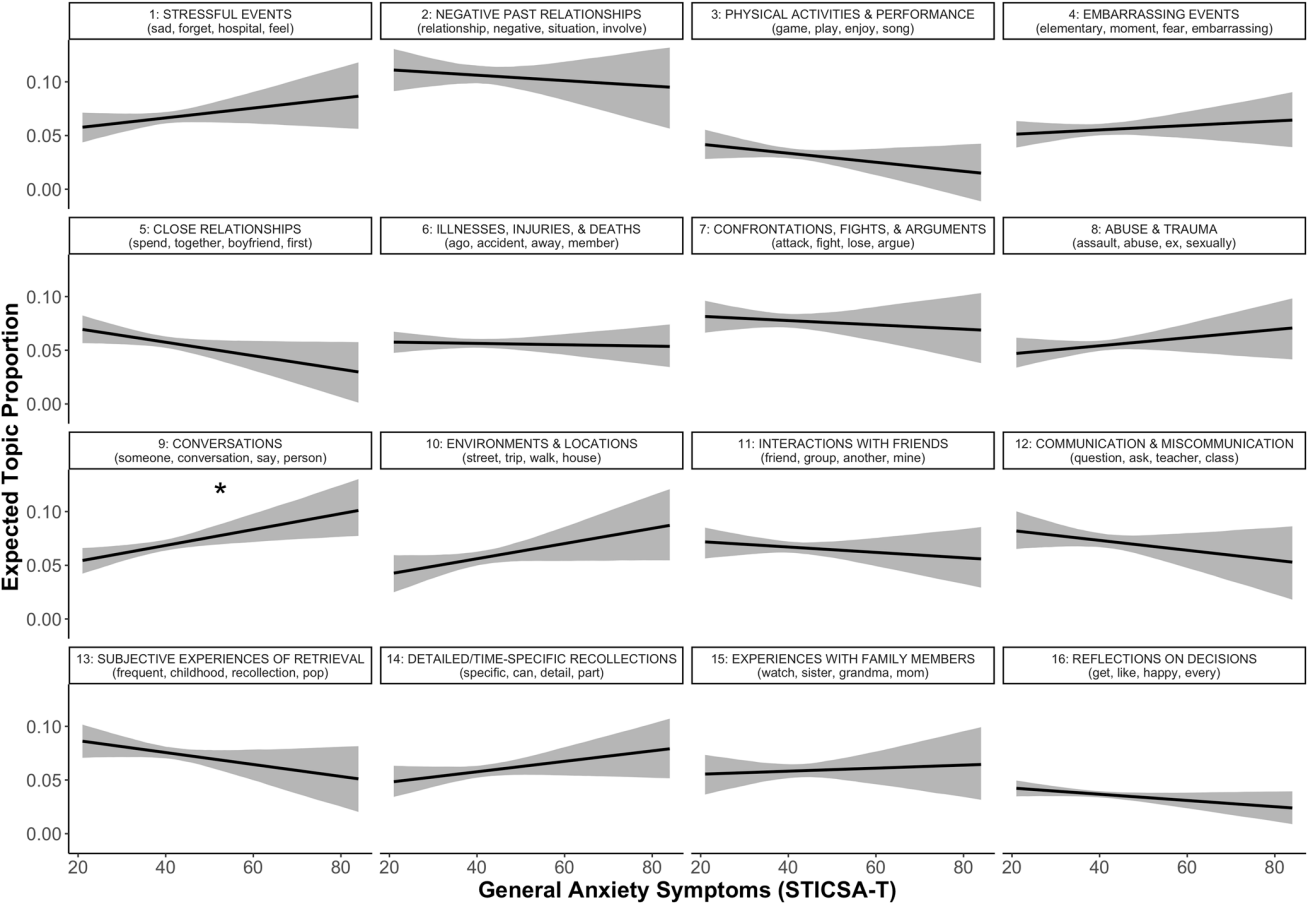
Predicted topic prevalence using general anxiety symptoms. STICSA-T State-Trait Inventory of Cognitive and Somatic Anxiety—Trait Version. Different panels represent different topics, denoted by topic numbers and most representative words at the top of each panel. *p < .05. Shaded ribbons represent 95% confidence intervals.

General anxiety symptoms were significantly and uniquely predictive of greater use of topic 9 (“Conversations”; Fig. 5).

## Discussion

Controversy surrounds the basic nature of recurrent IAMs. What are they typically about? Which of them—if any—are dysfunctional (i.e., related to worse mental health)? Some authors have speculated that only a subset of recurrent IAMs is maladaptive^8,9^. Evidence suggests that this maladaptive subset could be characterized by negative valence^3,4,51–54^. However, valence is potentially entangled with content (i.e., what people report remembering), since valence is related but dissociable from content. In other words, while some events or topics might typically be associated with certain valences (e.g., “Experiences with family members” and positive valence), content can also be relatively independent from valence. It remains relatively unexplored whether content (one’s reconstruction of the event) is related to psychopathology, after accounting for valence (emotional responses to IAMs). In the current study, we examined whether the content and valence of recurrent IAMs could differentiate between memories that are related to worse mental health status versus those that are not. By analyzing content in large samples of recurrent IAMs using structural topic modeling^15,42,43^, our work indicates that both the valence and content of one’s recurrent IAMs are linked to symptoms of mental health disorders.

Specifically, we replicated the relationship between negative IAM valence and greater symptoms of all disorders^3,4^. This result is consistent with past work showing more negative AMs in individuals with major depressive disorder^52^, PTSD^54^, social anxiety disorder^53^, or generalized anxiety disorder^51^. It also replicates a significant association between more negative AM valence and greater depression symptoms^55^. Interestingly, Rubin et al.^15^ also report a nonsignificant correlation between AM valence and PTSD symptoms (*r* = −.12). Here, we found significant negative correlations between recurrent IAM valence and depression, as well as PTSD symptoms, potentially because of the much larger sample size (and statistical power to detect effects). Alternatively, the current work may have found these effects because it focused on *recurrent* IAMs whereas Rubin et al.^15^ examined a variety of AMs (e.g., both voluntary and involuntary ones); it may be that involuntary or recurrent memories have stronger relationships to mental health than voluntary memories. The current results provide empirical support for theoretical models wherein recurrent IAMs, and the emotions they evoke, are a transdiagnostic process involved in psychopathology^5,7^, even in large, nonclinical samples.

In addition, our topic model allowed us to test hypotheses about whether one’s level of psychopathology is associated with recurrent memory content^2,3,5^. We used participants’ symptoms of mental health disorders (i.e., depression, PTSD, social anxiety, general anxiety) to predict the content (i.e., topic prevalence) within recurrent IAMs. What we have shown here is that some topics were seemingly benign: some topics such as “Physical activities and performance” and “Environment and locations” were not significantly related to any mental health index. Critically, specific topics were significantly associated with symptoms of *some* mental health disorders, but not others; no topics were universally related to symptoms of *all* mental health disorders (i.e., depression, PTSD, social anxiety, general anxiety), suggesting that recurrent IAM content is disorder-specific.

One of our key findings was that each mental health index uniquely predicted the prevalence of distinct topics. For example, while PTSD symptoms were significant positive predictors of the “Negative past relationships” topic (e.g., “relationship”, “negative”, “situation”, “traumatic”), depressive symptoms were significant positive predictors of the “Assaults and abuse” topic (e.g., “assault”, “abuse”, “trauma”, “fail”). Furthermore, social anxiety symptoms were significant positive predictors of the “Communication and miscommunication” topic (e.g., “question”, “ask”, “teacher”, “class”), and general anxiety symptoms were significant positive predictors of the “Conversations” topic (e.g., “someone”, “conversation”, “say”, “person”). These findings support the idea that there is indeed disorder-specific content in recurrent IAMs^6,16^, and that recurrent IAMs containing certain types of content are more likely to reflect psychopathology than other types of content^5,19^. In fact, we replicated a significant positive relationship between use of the “Assaults and abuse” topic and depression symptoms^19,56^, but with a much larger sample size and in a nonclinical sample. Our current evidence also lends support to hypotheses about the nature of emotional memory processes in PTSD. In particular, models of PTSD have suggested that difficulties in retrieving positive memories could underlie the development or maintenance of PTSD^57–60^. In line with these ideas, our results indicated that greater PTSD symptoms were related to less use of a positive topic (“Interactions with friends”) in recurrent IAMs. Topics significantly related to social anxiety (e.g., topic 12: “Communication and miscommunication”, e.g., incorrectly answering a question aloud during a class) seem to reflect ideas that recurrent IAMs in social anxiety disorder might focus on specific, aversive social events that individuals have experienced^53,61^. Similarly, topics significantly related to general anxiety (topic 9: “Conversations”) might reflect the high prevalence of social/interpersonal concerns as a worry topic in generalized anxiety disorder^62,63^.

Overall, our results suggest that while it is accurate to say that negative recurrent IAMs are consistently related to increased symptoms of psychopathology^3,4^, this statement can now be refined. Here, we show that both valence *and* specific types of content in recurrent IAMs are related to symptoms; self-reported valence as well as the use of specific topics in recurrent IAMs were significantly related to mental health indices. Moreover, many negative topics were not significantly related to symptoms of any disorder (e.g., “Stressful events”, “Confrontations, fights, and arguments”). Though emotions evoked by recurrent IAMs were an important component in the relationship between these memories and psychopathology, our study suggests that content (e.g., types of events described, how the individual reconstructs them) is also vital to consider and provides unique insight into mental health status. This is notable because it suggests that the emotional valence ascribed to specific memories is not entirely sufficient to distinguish maladaptive recurrent memories from benign ones. Based on our analyses, even if participants attributed the same level of valence to their memories (e.g., “very negative”), the way they reconstructed the memory (i.e., content) was still significantly related to their current symptoms of mental health disorders.

In terms of limitations, we only investigated recurrent IAMs—a specific type of AM—in the present study. While there is theoretical precedent claiming that these memories are particularly relevant to psychopathology^5,7^, the current results could be compared against voluntary AMs to test these previous hypotheses. For instance, it would be valuable to examine if there are dissociations or similarities between voluntary and involuntary AM content in relation to PTSD symptoms (as in^55^). Also due to the current study’s focus on recurrent IAMs, our data were largely limited to a past-focused temporal orientation, as participants were instructed to only describe/rate memories. While participants were free to describe future-oriented content, this would have been incidental (at the discretion of the participant) and related to their past-oriented memories. Future studies could compare memories to other forms of spontaneous or clinically relevant thought (such as episodic future thoughts, ruminations, or worries) to investigate how content and/or valence might have varying relationships with psychopathology depending on the type of thought. Alternatively, studies could qualitatively classify topics in terms of whether they are more past- or future-oriented.

Another relevant design choice was to focus on participants’ one most frequently recurring IAM. This approach is consistent with past literature^2–4^, which facilitates comparison with prior studies. However, past work has also found that participants report experiencing many different recurrent IAMs (*M* = 7.3^3^). As such, future studies could ask participants to list all (or at least multiple) recurrent IAMs they have experienced recently. By asking participants to describe and/or rate these less frequent recurrent IAMs, one could potentially better capture the variety of memories that individuals experience in their daily lives.

Additionally, it is worth noting that the cross-sectional nature of the current study also introduces limitations. Participants completed all measures in a single, asynchronous, online session. Potentially, any given participant could have been in a negative mood during the study, which could have led to greater accessibility of negative recurrent IAMs, or more negative ratings of these memories’ valence^1^. At the same time, negative mood could have inflated participants’ scores on mental health indices^64^. While mood is indeed part of theoretical models of why recurrent IAMs persist and correlate with mental health outcomes^29,65^, the current work cannot conclusively discern the directionality of effects observed. Future work could consider methods such as ecological momentary assessment^66^ to unravel temporal dynamics of recurrent IAMs and the emotions preceding/following them.

We also used a large, nonclinical convenience sample of undergraduates in the current study. While it can be assumed that these participants were overall nonclinical (e.g., not patients with psychological/psychiatric diagnoses), based on general base rates of psychopathology, it is probable that some number of our participants were experiencing clinically relevant mental health challenges. Future work could recruit large samples of clinical and/or nonclinical participants and potentially examine whether the current observations are consistent across populations. In a similar vein, the current study examined symptoms as a dimensional measure of psychopathology, rather than a categorical distinction (e.g., individuals with disorders vs. individuals without). It remains possible that psychopathology could have nonlinear relationships with recurrent IAMs that emerge when comparing those with or without diagnosed mental health disorders.

Finally, future work could extend the computational approach taken in the current study. Here, we used a unigram, bag-of-words approach of topic modeling, which is common in the natural language processing literature^67^. While this approach typically performs well and effectively captures content^41,42^, it has a few noteworthy drawbacks. Unigram approaches can miss multiword phrases (e.g., “family” and “member” being highly representative of topic 6, rather than “family member”), which could potentially make a topic difficult for researchers to interpret (e.g., seeing “member” without “family”). Moreover, the bag-of-words approach does not have access to information about word order^41^, which can sometimes change the meaning of a document (e.g., “is it good?” vs. “it is good”). Developments in natural language processing and associated software could enable future studies to incorporate these additional features into topic models.

In conclusion, our current study is the first to provide a comprehensive description of how both recurrent IAM valence *and* content predict symptoms of mental health disorders in a large, nonclinical sample. While recurrent IAM valence seems to have relatively homogenous relationships with symptoms across various disorders, recurrent IAM content appears to differentiate between disorders. By using machine learning and natural language processing techniques in a novel application (i.e., autobiographical memory), we present a robust and reproducible topic model that reveals unique relationships between specific topics and specific mental health indices. Our work shows that AM phenomenology (e.g., valence) and content can be analyzed in tandem, and at much larger scales than previously thought possible, to answer critical questions about the fundamental nature of recurrent IAMs. Topics in recurrent IAMs—and their links to mental health—are identifiable, distinguishable, and quantifiable.

### Supplementary Information

Figure S1

## Data Availability

The datasets generated and/or analyzed during the current study are available on the Open Science Framework (10.17605/OSF.IO/GUR5V).

## Code availability

The underlying code for this study is available on the Open Science Framework (10.17605/OSF.IO/GUR5V).
