# Supplementary material for: Specific topics, specific symptoms: linking the content of recurrent involuntary memories to mental health using computational text analysis
Source: Npj Ment Health Res. 2023 Dec 18;2:22. doi: 10.1038/s44184-023-00042-x (PMC10955861; doi:10.1038/s44184-023-00042-x)
Supplement: Supplementary file 1 — Figure S1 [file 44184_2023_42_MOESM1_ESM.docx]

**Figure S1**

*Histograms of Mental Health Indices*


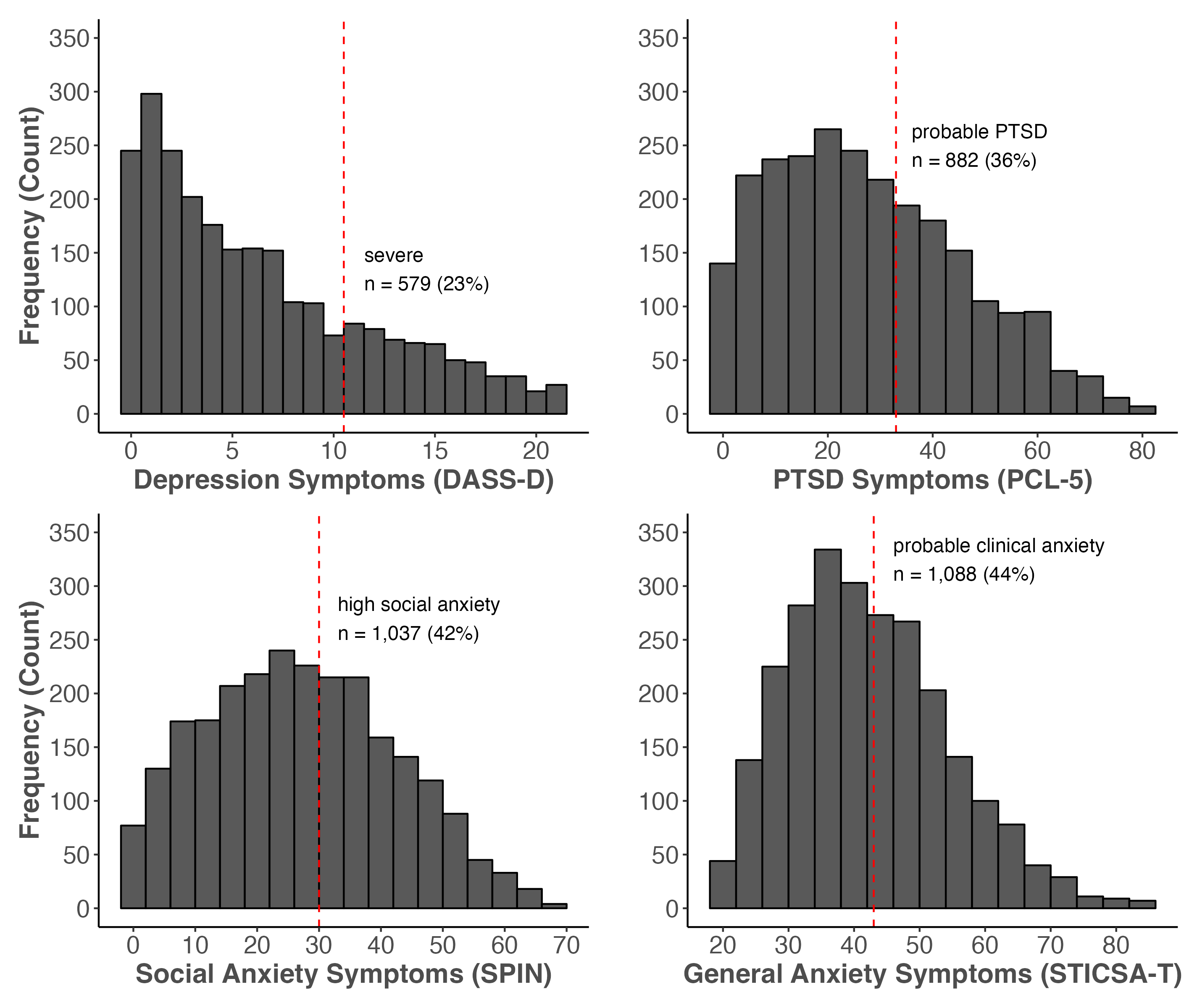


*Note.* DASS-D = Depression, Anxiety, Stress Scales – Depression Subscale, PTSD = posttraumatic stress disorder, PCL-5 = PTSD Checklist for DSM-5, SPIN = Social Phobia Inventory, STICSA-T = State-Trait Inventory of Cognitive and Somatic Anxiety – Trait Subscale. Cutoff scores are based on previous work (Lovibond & Lovibond, 1995; Moscovitch et al., 2012; Van Dam et al., 2013; Weathers et al., 2013).
